# Bile acids and bile acid modification in health and disease: from novel modifications to therapeutic interventions

**DOI:** 10.3389/fendo.2026.1837504

**Published:** 2026-06-04

**Authors:** Chuying Yu, Xintong Ye, Haoyu Zhao, Suting Qian, Feifei Xie, Qingsheng Liu

**Affiliations:** 1 Department of Gastroenterology Zhejiang Chinese Medical University, Hangzhou, China; 2 Department of Gastroenterology, The Third People's Hospital of Hangzhou, Hangzhou, China

**Keywords:** bile acid modifications, bile acids, chemical modification, farnesoid X receptor, novel modifications

## Abstract

Bile acids (BAs) serve dual roles as lipid-digesting molecules and key signaling mediators in metabolism and immune regulation. This review systematically examines: 1.the molecular mechanisms underlying BA-mediated regulation of glucolipid m5etabolism and energy expenditure; 2.host- and microbiota-driven BA modifications (e.g., 3-acylation); 3. therapeutic targeting of BA signaling pathways (FXR/TGR5). We highlight emerging strategies, including novel BA modifications, microbiota interventions, and BA-targeted therapies, which reshape BA homeostasis and show therapeutic potential for metabolic, digestive, hepatobiliary, and neoplastic diseases. Furthermore, we emphasize the central role of BA modifications in metabolic regulation and their pathological implications. Advances in multi-omics and AI-driven approaches deepen mechanistic insights and accelerate the translation of BA-based interventions into clinical practice. Most of the findings discussed in this review are derived from preclinical studies (*in vitro* and animal models); clinical translation of bile

## Introduction

Bile acids (BAs), classically recognized as emulsifiers of dietary lipids, have emerged as pivotal signaling molecules governing metabolic homeostasis, immune regulation, and gut microbial ecology. Synthesized in the liver from cholesterol and further modified by gut microbiota, BAs exhibit remarkable structural diversity through host enzymatic pathways (e.g., hydroxylation, conjugation) and microbial biotransformations(e.g.,deconjugation,7α-dehydroxylation) (1). These modifications critically determine their receptor-binding affinities and biological activities, enabling BAs to regulate key nuclear receptors (e.g., farnesoid X receptor, FXR) and membrane receptors (e.g., Takeda G protein-coupled receptor 5, TGR5) (2).

Beyond their classical roles in synthesis and enterohepatic circulation, bile acids (BAs) critically regulate macronutrient metabolism (lipids, carbohydrates, proteins) and systemic inflammatory balance (3). Emerging evidence indicates that microbiota-driven BA modifications (e.g., 3-acylation, polyamine conjugation) not only enhance BA diversity but also mechanistically link gut dysbiosis to metabolic disorders, inflammatory pathologies, and carcinogenesis (4). For instance, 3-succinylated cholic acid (3-sucCA), a novel microbial metabolite, alleviates metabolic dysfunction-associated steatohepatitis (MASH) by enhancing intestinal barrier integrity (5). However, key unanswered questions persist: (1) how organ-specific BA modifications regulate immune crosstalk, and (2) whether precision engineering of BA pathways can mitigate adverse effects (e.g., hepatotoxicity of FXR agonists) while retaining therapeutic efficacy (5).

This review synthesizes cutting-edge insights into BA modification mechanisms, their pathological implications, and therapeutic innovations. We highlight breakthroughs in click chemistry-driven metabolite discovery, AI-aided drug design, and microbiota-targeted interventions, offering a roadmap for translating BA biology into clinical applications.

## Bile acid homeostasis, modifications, and signaling mechanisms

### Molecular mechanisms of bile acid synthesis and modification

Depending on the source, BA can be divided into primary and secondary BA. In hepatocytes, the BA that is synthesized directly from cholesterol is known as primary BA, which includes chenodeoxycholic acid (CDCA) and cholic acid (CA). After the primary synthesis of BA, the majority of BAs are converted from the free to the bound state by binding to taurine or glycine; BAS form sodium salts at the physiological pH, which increases their solubility (6). Primary BAs enter the intestine and are converted by the enzymatic activity of intestinal bacteria into secondary BAs, mainly deoxycholic acid (DCA) and trace amounts of lithocholic acid (LCA) (7). These total BAs circulate in the body’s enterohepatic circulation, forming a BA pool that includes the liver (<1%), intestine (85% to 90%) and gallbladder (10% to 15%) (8). Once bile acids are synthesized, bound and secreted into the intestine, approximately 95% are reabsorbed in the terminal ileum and returned to the liver.

#### Host synthesis pathway

**Figure 1:**
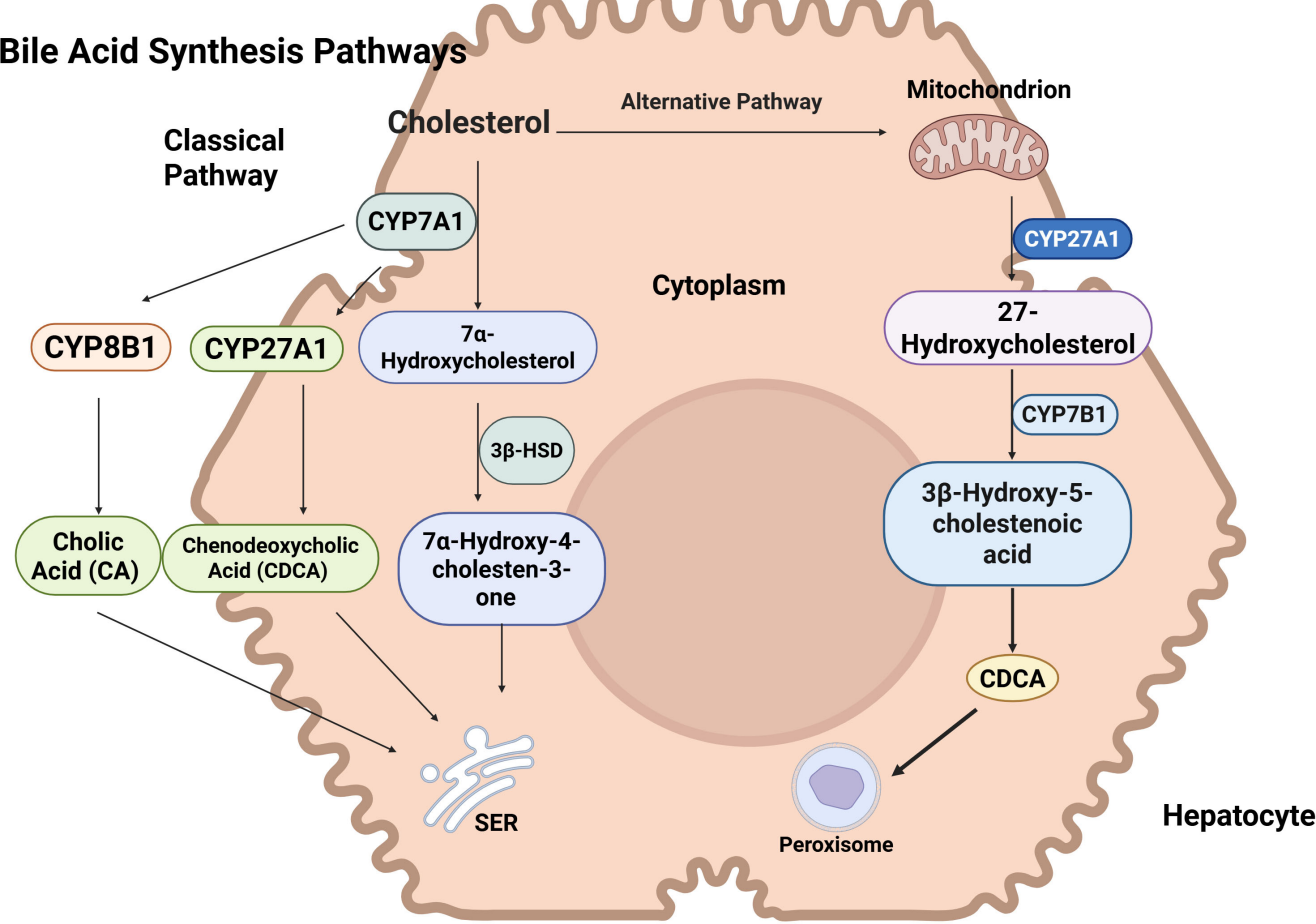
Schematic diagram of the two primary bile acid synthesis pathways in hepatocytes. Cholesterol is converted into primary bile acids, cholic acid (CA) and chenodeoxycholic acid (CDCA), via the classical (neutral) and alternative (acidic) pathways. The classical pathway, initiated by the rate-limiting enzyme CYP7A1, produces both CA and CDCA. The alternative pathway, initiated by CYP27A1, primarily produces CDCA. Key intermediates and enzymes (e.g., 3β-HSD, CYP8B1) are indicated. SER, smooth endoplasmic reticulum.

As illustrated in **Figure 1**. Bile acid synthesis in the liver follows two routes: the classical pathway (initiated by CYP7A1, accounting for ~75% of production) and the alternative pathway (initiated by CYP27A1). The classical pathway produces both cholic acid (CA) and chenodeoxycholic acid (CDCA), while the alternative pathway primarily yields CDCA. Key enzymes include CYP8B1 (determining CA/CDCA ratio), CYP27A1, and CYP7B1. After synthesis, bile acids are conjugated with glycine or taurine before secretion. 12α-hydroxylated bile acids (e.g., CA) beneficially regulate glucose and lipid metabolism, partly through FXR-dependent and independent mechanisms (9, 10) (1, 11).

#### Bacterial community modification

##### Deconjugation

**Figure 2:**
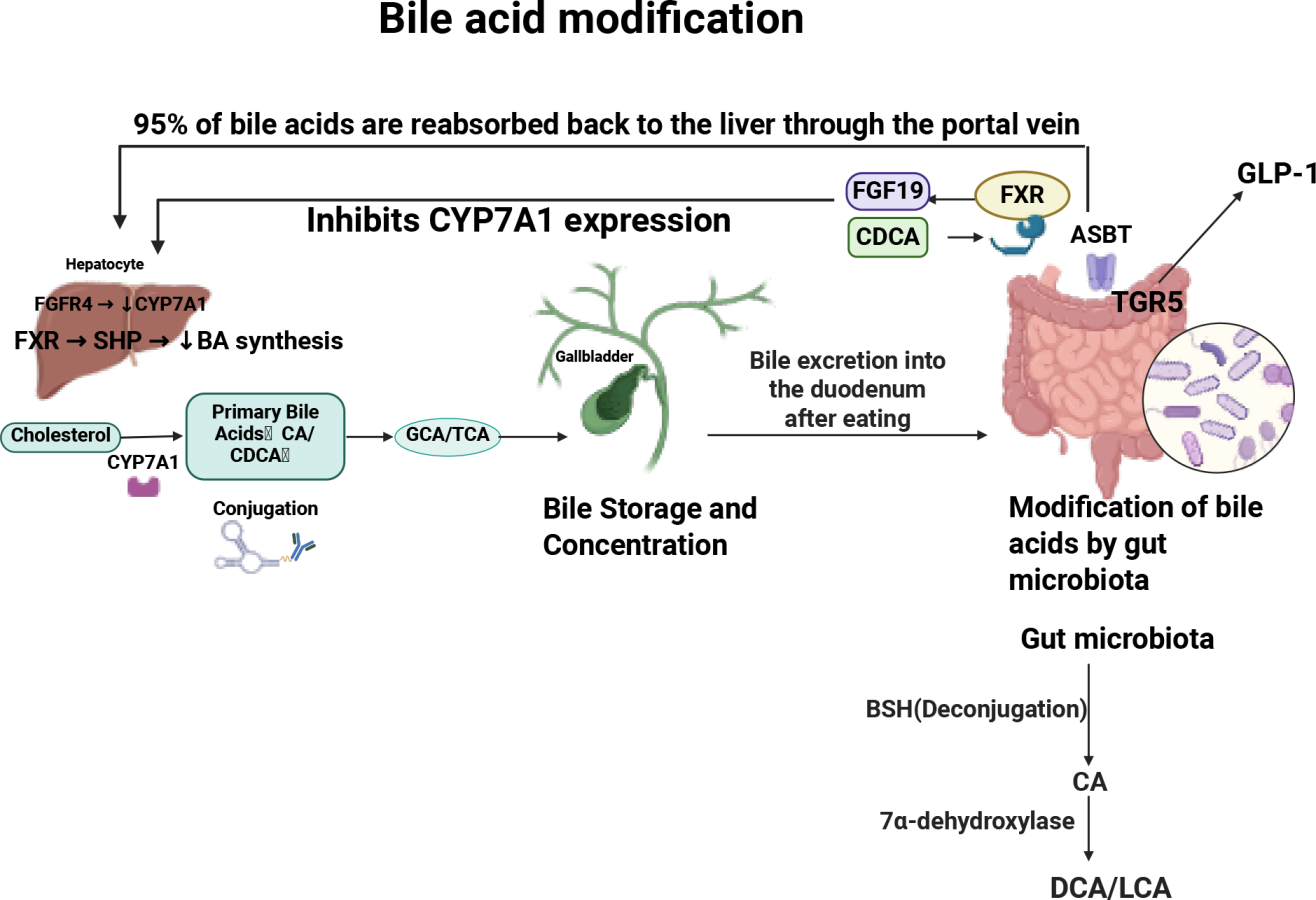
The enterohepatic circulation and gut microbiota-mediated modification of bile acids. The enterohepatic circulation and gut microbiota-mediated modification of bile acids. Primary bile acids (CA, CDCA) are conjugated, stored in the gallbladder, and secreted into the intestine. Approximately 95% are reabsorbed in the ileum via ASBT and returned to the liver. In the intestine, gut microbiota perform deconjugation (BSH) and 7α-dehydroxylation. Specific bacteria responsible for 7α-dehydroxylation (e.g., Clostridium scindens, C. hylemonae) are indicated. Reabsorbed bile acids activate FXR in enterocytes, leading to FGF15/19 secretion, which acts on liver FGFR4 to suppress CYP7A1 (FXR–FGF15 pathway). In hepatocytes, FXR also activates SHP to repress bile acid synthesis (FXR–SHP pathway). In intestinal L cells, TGR5 activation stimulates GLP-1 secretion (TGR5–GLP1 pathway). The clinical relevance of the TGR5-GLP1 axis is discussed in the context of current GLP-1 receptor agonists. These signaling cascades coordinate bile acid homeostasis and metabolic regulation.

Bile acid dissociation is carried out by bacteria with bile salt hydrolase (BSH) activity (**Figure 2**), which acts as a catalyst for the hydrolysis of the amide bond to produce unbound bile acids, and microbial dissociation (i.e., removal of glycine or taurine couplings) prevents the active reuptake of these bile acids via the apical sodium-dependent bile acid transporter protein (ASBT) (1). BSH is not only the basis for dissociation, but is also a prerequisite for 7α-dehydroxylation, which results in the production of secondary bile acids. BSH activity is implicated in lipid metabolism and weight gain (12), immune function (13) and circadian rhythms (14). BSH function is common to all major bacterial branches in the gut (15).Microbial bile acid dissociation is beneficial to gut microbial populations as it is associated with the acquisition of glycine and taurine for further metabolism to produce carbon, nitrogen, and sulfur for growth. Changes in gut microbial populations resulting in altered levels of BSH expression will have an impact on the detergent and signaling properties of the bile acid pool (16).

##### Dehydroxylation

The 7α-dehydroxylation reaction is carried out by multiple enzymes encoded by the Bai (Bile Acid Inducible) operon in a synergistic manner under an anaerobic environment. BaiA2 and BaiB catalyze the oxidation of the 3α-hydroxyl group of CA to the 3-keto intermediate, while consuming NAD+ to generate NADH; then, BaiCD (a ferric thioflavonoid enzyme) catalyzes the formation of the steroid A ring to form the Δ4,5 double bond, generating the intermediate Δ4-3-ketocholic acid; then, BaiH (an NADH-dependent reductase) reduces the Δ4,5 double bond to a single bond, generating the intermediate isocholic acid; finally, BaiE and BaiF synergistically catalyze the elimination of the 7α-hydroxyl group (17),ultimately generating the secondary bile acids DCA (from CA) or LCA (from CDCA), which are the most abundant microbial bile acids metabolized in human feces (16). The entire 7α-dehydroxylation pathway is encoded by the bile acid inducible (bai) operon, which includes genes baiA through baiI. This operon is present in specific gut bacteria such as Clostridium scindens and Clostridium hylemonae, enabling the conversion of primary bile acids (CA and CDCA) to secondary bile acids (DCA and LCA) (17, 18).

Microbial 7α-dehydroxylation catalyzed by the bai operon represents a critical enzymatic transformation that profoundly remodels the hydrophobicity index of the bile acid pool, a key physicochemical parameter governing biological activity and host metabolism. Primary bile acids synthesized in the liver, such as cholic acid (CA) and chenodeoxycholic acid (CDCA), are relatively hydrophilic due to the presence of multiple hydroxyl groups. During intestinal transit, anaerobic bacteria including Clostridium scindens and Clostridium hylemonae remove the 7α-hydroxyl group via 7α-dehydroxylation, converting CA into deoxycholic acid (DCA) and CDCA into lithocholic acid (LCA). This single structural modification drastically increases bile acid hydrophobicity: the hydrophobicity index rises from hydrophilic (CA/CDCA) to moderately hydrophobic (DCA) and highly hydrophobic (LCA) (19).

This physicochemical shift directly dictates host metabolic responses through multiple mechanisms. First, hydrophobic bile acids exhibit stronger membrane-intercalating capacity, disrupting intestinal epithelial tight junctions, increasing gut permeability, and promoting translocation of lipopolysaccharide (LPS) into the circulation, which triggers chronic low-grade inflammation, insulin resistance, and hepatic steatosis. Second, hydrophobic bile acids display receptor signaling bias: they preferentially activate the membrane receptor TGR5 while attenuating FXR activation. TGR5 hyperactivation enhances energy expenditure and GLP-1 secretion, but sustained hydrophobic bile acid overload drives pro-inflammatory signaling and oxidative stress. Third, highly hydrophobic bile acids are cytotoxic, inducing endoplasmic reticulum stress, DNA damage, and hepatocyte injury, which exacerbate metabolic dysfunction-associated steatohepatitis (MASH) and increase gastrointestinal cancer risk. Collectively, microbial 7α-dehydroxylation reshapes bile acid hydrophobicity, which acts as a central physicochemical switch that integrates gut microbiota activity with systemic metabolic homeostasis and disease susceptibility (20, 21).

##### Sulfation

Sulfation begins when the hydroxyl or carboxylic acid group of a bile acid (e.g., lithocholic acid LCA, deoxycholic acid DCA) is recognized by sulfotransferase, followed by the sulfotransferase transferring the sulfate group from the PAPS to the bile acid, forming a sulfate bond to produce sulfated bile acids (e.g., LCA-3S, DCA-3S), which have increased aqueous solubility and decreased toxicity (22).

The enterohepatic circulation of bile acids (~95% reabsorption in the ileum) ensures their efficient recycling and allows microbiota to modify a significant fraction of the bile acid pool (23).

#### Novel modification discovery

Beyond canonical deconjugation and 7α-dehydroxylation, gut microbiota drive unconventional structural modifications that expand bile acid chemical diversity and rewire host signaling, representing the innovative core of bile acid biology. These modifications, including C3-O-acylation, C24-amidation, and polyamine conjugation, are catalyzed by specific commensal bacteria and exert distinct metabolic and immunomodulatory effects (24).

C3-O-acylation is a landmark microbial modification, catalyzed by Anaerobacterium spp. (via the β-lactamase homolog BAS-suc) and Christensenella minuta. Short-chain fatty acids (e.g., succinate, butyrate) or long-chain fatty acids (e.g., palmitate) esterify the C3 hydroxyl group of primary bile acids, forming 3-acylated bile acids (e.g., 3-succinylcholic acid, 3-sucCA). This structural change confers unique receptor bias: 3-sucCA preferentially activates TGR5 while exhibits reduced agonism for FXR, shifting signaling toward metabolic benefits. Preclinical data show 3-sucCA enhances intestinal barrier integrity, promotes GLP-1 secretion, alleviates hepatic steatosis, and improves insulin sensitivity in diet-induced obese mice, with minimal FXR-related adverse effects (e.g., pruritus, gallbladder dysfunction) (25).Although head-to-head human receptor data remains limited, these modifications show emerging promising receptor bias and gut-restricted properties, minimizing systemic off-target effects.

C24-amidation represents another critical modification, where gut microbiota conjugate bile acids at the C24 carboxyl group with diverse amino acids (e.g., alanine, glutamine, tryptophan), generating over 200 amino acid-bile acid conjugates. Unlike classical glycine/taurine conjugation, this process relies on non-BSH acyltransferase activity (26, 27). C24-amidated bile acids exhibit tissue-specific immunomodulation: they selectively regulate T cell differentiation, suppress intestinal inflammation, and mitigate inflammatory bowel disease (IBD) pathology. Some conjugates also enhance bile acid solubility and reduce cytotoxicity, protecting the gut epithelium (24).

The attachment of acyl groups (C3-O-acylation) or amino acids (C24-amidation) alters the polarity, solubility, and resistance to enzymatic degradation of bile acids. For example, 3-succinylation increases aqueous solubility and reduces passive diffusion across membranes, potentially confining its action to the gut lumen and portal circulation. In contrast, C24-amidation with bulky aromatic amino acids (e.g., phenylalanine) may enhance binding to TGR5 while reducing FXR affinity, creating biased signaling. These modifications also protect bile acids from deconjugation by BSH, prolonging their half-life in the enterohepatic circuit. The resulting changes in receptor selectivity and metabolic stability offer opportunities for designing next-generation bile acid-based therapeutics with fewer off-target effects (24).

Polyamine conjugation (e.g., putrescine-CA, spermidine-CDCA) is a rare but functionally potent modification. Catalyzed by specific gut anaerobes, these conjugates selectively modulate TGR5 signaling, exert robust anti-inflammatory effects, and inhibit macrophage pro-inflammatory cytokine release. Although present at low abundance, polyamine-conjugated bile acids show promising potential for treating chronic inflammatory disorders (28).

Collectively, these novel microbial modifications redefine bile acid biology: they not only expand the bile acid pool diversity but also serve as key mediators of gut microbiota-host crosstalk. Targeting these modifications—via microbiota interventions, enzyme modulators, or synthetic analogs—offers a promising strategy to restore bile acid homeostasis and treat metabolic, inflammatory, and neoplastic diseases, bridging basic research and clinical translation.

The major types of microbiota-driven bile acid modifications, their responsible enzymes and microbial taxa, as well as their functional implications, are summarized in **Table 1**.

**Table 1:** Types of microbiota-driven bile acid modifications, key enzymes/microbes, and functions.

Modification type	Key enzyme/gene	Representative microbial taxa	Function/pathological implication
Deconjugation	Bile salt hydrolase (BSH)	*Lactobacillus*, *Bifidobacterium*, *Bacteroides*	Releases free bile acids; affects lipid metabolism and immunity
7α-dehydroxylation	*bai* operon (BaiA–I)	*Clostridium scindens*, *Clostridium hylemonae*	Produces secondary bile acids DCA and LCA
Sulfation	Sulfotransferase (SULT; host-derived)	(Microbiota indirectly regulate)	Increases solubility, reduces toxicity
C3-O-acyl esterification	BAS-suc (β-lactamase homologue)	*Anaerobacterium*, *Christensenella minuta*	Generates short-chain fatty acid-bile acid esters; metabolic regulation
C24-amidation	BSH acyltransferase activity (or novel enzymes)	Multiple gut bacteria	Produces amino acid-conjugated bile acids; immune modulation

#### Bile acid receptor FXR and TGR5: dual core targets for metabolic regulation and disease therapy (**Figure 2**)

The main BA-mediated nuclear receptors are farnesoid X receptor (FXR), pregnane X receptor and vitamin D receptor (29).

FXR is mainly expressed in the intestine and liver (30). FXR is potently activated by various bile acids, with chenodeoxycholic acid (CDCA) being one of its most potent endogenous agonists, and regulates lipogenesis and triglyceride synthesis (31). FXR signaling may have a variety of physiological roles, in addition to negative feedback regulation of BA synthesis and transport, and modulation of the immune response and energy metabolism (32). Numerous studies have demonstrated that Post-translational modifications of FXR (PTM) have an effect on the mechanism of action of diseases (33).

In terms of cell membrane receptors, BAS primarily recognizes Takeda G-protein-coupled receptor 5 (TGR5, also known as GPBAR1) (34) (**Figure 2**). In addition to TGR5, G protein-coupled receptors activated by BA include sphingosine-1-phosphate receptor 2 (35).TGR5 is minimally expressed in healthy hepatocytes but is highly abundant in Kupffer cells, biliary epithelial cells, intestinal L-cells, and immune cells (36).

FXR and TGR5 are two of the most important BA-mediated receptors. Their signaling enables crosstalk between the gut and the liver. The interaction between FXRs and TGR5s is key to lipid metabolism and protection against inflammatory diseases such as NAFLD, diabetes and atherosclerosis. Selective ligand activation of FXR and TGR5 diminishes hepatic inflammation and injury, diet-induced obesity, insulin resistance, and atherosclerosis by decreasing adipogenesis and gluconeogenesis. Bile acid (BA)-FXR and ba-TGR5 signaling are broadly engaged in hepatocellular carcinoma pathogenesis. Therefore, the development of FXR and TGR5 modulators suggest a potential basis for future therapeutic strategies in hepatocellular carcinoma, pending further preclinical and clinical evaluation (29).

#### TGR5–GLP-1 axis and its therapeutic synergy with current incretin-based therapies

The TGR5–GLP-1 axis has emerged as a critical signaling node linking bile acid metabolism to systemic glucose homeostasis and energy expenditure (37). While canonical bile acids (e.g., CA, CDCA, DCA) can activate both FXR and TGR5, their broad receptor engagement often leads to undesirable off-target effects, including hepatic toxicity, pruritus, and gallbladder dysfunction. In contrast, novel microbiota-driven bile acid modifications, particularly C3-O-acylation and C24-amidation, exhibit distinct receptor signaling bias, preferentially activating TGR5 while attenuating FXR agonism (38). For example, 3-succinylated cholic acid (3-sucCA), a representative C3-acylated bile acid, displays enhanced potency toward TGR5 in intestinal L-cells, triggering robust GLP-1 secretion with minimal FXR-mediated hepatic feedback. Similarly, C24-amidated bile acids selectively modulate TGR5 signaling, promoting anti-inflammatory responses and improving insulin sensitivity without disrupting bile acid homeostasis (39).

As endogenous GLP-1 secretagogues, these modified bile acids offer unique mechanistic advantages over canonical bile acids. First, their TGR5-biased profile minimizes adverse effects associated with FXR hyperactivation, such as dyslipidemia and gallstone formation. Second, they enhance intestinal barrier integrity and reduce local inflammation, creating a favorable microenvironment for sustained GLP-1 production. Third, microbial-derived modified bile acids are dynamically regulated by gut microbiota, enabling physiological adaptation to metabolic stress. Preclinical studies have demonstrated that 3-sucCA supplementation increases circulating GLP-1 levels, improves glucose tolerance, and reduces hepatic steatosis in diet-induced obese mice, as endogenous GLP-1 secretagogues (40).

### Bile acid modification in disease and therapy

#### Inflammatory bowel disease

The apical sodium-dependent bile acid transporter protein (ASBT) in the ileum is essential in the reabsorption of BA in the enterohepatic circulation. Therefore, reduced ASBT function impairs intestinal absorption, leading to increased excretion of BAs in the feces and bile acid malabsorption (BAM), which is common in patients with IBD (41). Proinflammatory cytokines such as interleukin (IL)-1β and TNF, which inhibit the activity of the ASBT promoter, may be a reason for the lower expression of ASBT in patients with IBD (42). Typically, Crohn’s disease (CD) involves the ileum, which is the main site of ASBT expression, so the extent of BAM may be more severe in CD patients with a history of ileal resection (43).

FXR modulates BA generation with feedback inhibition of CYP7A1 via FXR/fibroblast growing element 19/hepatic fibroblast growth factor receptor 4 signaling (44, 45).Thus, BAM caused by defective ASBT function in CD and ulcerative colitis (UC) induces a decrease in the BA pool of intestinal epithelial cells, a reduction in the inhibition of FXR, and a subsequent rise in the production of BAs (46).

Changes in BA modification capacity due to bacterial dysbiosis could be one of the important factors in the development or progression of IBD (47). Modifications of BA, such as dissociation and conversion, are conducted by a wide range of intestinal bacteria. Studies have shown that the dissociative, transforming, and desulfurizing activities of the microbiota of IBD patients have been proven to be impaired (47, 48). In addition, the 7α-dehydroxylation enzymatic reaction is inhibited below pH 6.5. The pH of the feces of patients with Crohn’s disease has been shown to be acidic (49).

There are 2 broad types of BA therapies: displacement and replacement (19).In replacement therapies, it is likely to be useful to appropriately inhibit the synthesis of BA for increased PBA (Primary Bile Acid) and SBA (Secondary Bile Acid) and to use BA binders to facilitate their elimination. In alternative therapies, supplementation with SBA to correct BA deficiency may be of interest; SBA supplementation has been shown to be effective in reducing intestinal inflammation in 3 mouse models of colitis (46).

#### Metabolic diseases

##### Obesity

FXR can regulate lipid and lipoprotein metabolism by affecting lipoprotein secretion, hepatic lipogenesis, intravascular remodeling, and intestinal cholesterol uptake in a manner consistent with plasma clearance. By inhibiting hepatic SREBP 1c expression in a SHP (50) and GF 15/19-dependent manner, FXR can decrease lipogenesis. FXR also inhibits microsomal triglyceride transfer protein (STP) and apolipoprotein (apo) B gene expression thereby decreasing VLDL secretion (51).In addition to this, FXR enhances lipoprotein lipase (LPL) activity by increasing the expression of apoC-II (an LPL activator) and repressing apoC-III (an LPL inhibitor), thereby promoting intravascular lipolysis and triglyceride clearance (52, 53). Furthermore, FXR increases VLDL receptor (VLDLR) expression (54).

##### Diabetes, insulin resistance

BA works directly on FXR and TGR 5 in the intestine, liver and pancreas and indirectly regulates glucose homeostasis through facilitating FXR-dependent induction of intestinal GF 15/19. In the intestine, FXR modulates glucose uptake kinetics. FXR decreases postprandial glucose utilization by inhibiting hepatic glucose catabolism and lipogenesis (55), whereas GF 15/19 increases gluconeogenesis (56).

Both FXR and TGR5 are expressed in pancreatic β-cells and α-cells, mediating intra-islet paracrine crosstalk between insulin and glucagon regulation (57).Besides, TGR 5 activation in pancreatic a-cells induces the expression of convertase precursor 1, which switches 16 glucagon production to GLP-1, thereby enhancing β-cell mass and function in a paracrine way (58). Altered hepatic glucose metabolism affects BA synthesis and influences FXR-regulated glucose-stimulated insulin secretion from β-cells, thus defining the hepatic-pancreatic BA signaling link (59).

BAS treatment also improves glucose homeostasis, and colesevelam is an FDA-approved oral antidiabetic drug. Long-term treatment with BCA inactivates FXR in intestinal L-cells, enterocytes, and hepatocytes. It increases GLP-1 synthesis and secretion, reduces intestinal glucose absorption, enhances hepatic glucose catabolism and lipogenesis, thereby promoting visceral glucose utilization (60).

The rapid clinical success of GLP-1 receptor agonists (e.g., semaglutide, tirzepatide) has revolutionized the management of obesity and type 2 diabetes mellitus (T2DM), yet these agents remain limited by parenteral administration, high cost, gastrointestinal intolerance, and post-treatment weight regain. Bile acid-targeted modulators represent a promising complementary strategy, offering synergistic benefits when combined with current incretin therapies. Modified bile acids upregulate intestinal TGR5 expression and amplify endogenous GLP-1 secretion, thereby sensitizing tissues to exogenous GLP-1 agonists. This synergistic interaction enhances weight loss, improves insulin resistance, and allows dose reduction of GLP-1 drugs, mitigating side effects (61). Moreover, bile acid-based interventions may address the underlying dysbiosis and metabolic inflammation that drive T2DM progression, offering a disease-modifying approach rather than mere symptomatic control. Collectively, the integration of novel bile acid modification research with incretin pharmacology opens new avenues for developing safe, effective, and sustainable combination therapies for obesity and T2DM (62).

#### Non-alcoholic fatty liver disease

Two receptors, nuclear farnesoid X receptor (FXR) and membrane Takeda G protein-coupled receptor 5 (TGR5), are expressed on cells both along and outside the enterohepatic cycle.BA-induced activation of the FXR in the intestinal epithelium stimulates the production of FGF19, a critical component of enterohepatic FXR signaling (63). When FXR in intestinal epithelial cells is activated by BA, it produces FGF15, which is released in portal blood and stimulates the hepatic FGFR4-β Klotho complex, thereby inhibiting hepatic BA synthesis (63). In hepatocytes, BA also activates the FXR to suppress BA synthesis in an SHP-dependent way (64). Hence, low FXR stimulation and increased BA synthesis can occur through classical pathways.

FXR coordinates BA metabolism, and both FXR and TGR5 regulate lipid and glucose homeostasis, energy expenditure, inflammation, and fibrosis (65, 66). BA disturbance results in reduced BA signaling through FXR and TGR5, which affects BA, glucose and lipid metabolism, and inflammatory responses. Correcting BA composition restores FXR and TGR5 signaling, relates metabolic disorders and may help to attenuate NASH progression in preclinical models, although clinical validation is still needed. FXR and TGR5 modulate glucose and lipid metabolism, as well as inflammation. Thus pharmacological agonists could be considered for the treatment of obesity, type 2 diabetes mellitus, and NASH (5, 67). NASH treatment by targeting BA receptors via FXR, TGR5 or dual agonists leads to promising results but may be accompanied by side effects such as decreased HDLc, increased cholesterol, LDLc, itching, and gallbladder volume (5).

Insulin resistance also affects the metabolism of BAs as a risk factor and key element in the pathogenesis of NAFLD. It has been shown that proper insulin signaling inhibits FoxO1, an activator of the sterol 12α-hydroxylase CYP8B1, and reduces the synthesis of 12α-hydroxylated CA (68).

Since any modification of the size and composition of the BA pool will regulate TGR5 and FXR signaling and possibly influence NAFLD pathogenesis, alterations in enterohepatic BA contribute significantly to the development of NASH. Fully exploring the patterns of change in the enterohepatic BA pool, metabolism, and signaling in patients may be key to the development of effective therapeutic approaches without side effects (69).

#### Cancer

##### Hepatocellular carcinoma

Dysregulated bile acid signaling via FXR and TGR5 is implicated in HCC pathogenesis (20). FXR activation suppresses hepatocarcinogenesis by inhibiting NF-κB and Wnt/β-catenin pathways, while TGR5 signaling may exert context-dependent pro- or anti-tumor effects. Accumulation of hydrophobic bile acids (e.g., deoxycholic acid) induces DNA damage, endoplasmic reticulum stress, and chronic liver inflammation, creating a pro-tumorigenic microenvironment. Therefore, modulating bile acid composition or targeting FXR/TGR5 represents a potential chemopreventive or therapeutic strategy for HCC (70, 71).

##### Gastric cancer

BAs may possibly be associated with precancerous lesions of the gastric mucosa. Measurement of intragastric BAs can reflect the injury and severity of duodenogastric reflux, and high levels of BAs are linked to an increased risk of intestinal epithelial metaplasia (IM) (72, 73). Bile reflux grading was positively correlated with the severity of gastric mucosal lesions (74). Serum total bile acids (TBA) levels were positively correlated with the risk of GC. And patients with GC had significantly higher concentrations of BAs (75). In addition, preoperative serum TBA-positive patients tend to have lymph node metastasis, suggesting that BAs may be associated with GC lymph node metastasis and indicates its potential utility as a predictive marker for GC lymph node metastasis (76). In summary, BAs are positively related to the onset and progression of GC. Both IM patients and GC patients have significantly increased concentrations of BAs, which are positively correlated with disease severity (77).

BAs have dual roles in gastric cancer development, which may either inhibit tumors through anti-inflammatory or pro-apoptotic mechanisms, or directly promote gastric cancer cells through activation of specific signaling pathways (e.g., NF-κB, Wnt/β-catenin, etc.) or induction of Epithelial-Mesenchymal Transition (EMT) malignant progression (78).

Activation of NF-κB signaling pathway: secondary bile acids (e.g., DCA, LCA) activate NF-κB signaling by binding to TLR4 or generating reactive oxygen species (ROS), promoting the expression of pro-inflammatory factors (IL-6, IL-8) and anti-apoptotic proteins (Bcl-2), and enhancing cancer cell survival (79). Helicobacter pylori (HP) infection synergistically up-regulates NF-κB with BA, accelerating gastric cancer progression (High expression of NF-κB in HP+ gastric cancer is associated with metastasis.) Studies have shown that NF-κB expression is significantly elevated in HP-positive gastric cancer and correlates with tumor infiltration and metastasis (80).

Driving epithelial-mesenchymal transition (EMT): BA has been reported to promote invasive metastasis in gastric cancer cell lines by inhibiting glycogen synthase kinase 3β (GSK3β) activity, stabilizing β-catenin proteins, promoting their intranuclear accumulation, up-regulating EMT markers (e.g., Snail, Vimentin) and inhibiting E-cadherin expression; or alternatively, matrix metalloproteinases (MMPs) induction, BA upregulated the expression of MMP-2, MMP-7 and MMP-9 through NF-κB and AP-1 pathways, degraded the extracellular matrix and promoted gastric cancer cell invasion. For example, increased MMP-7 expression in DCA-treated gastric cancer cells correlated with clinical gastric cancer metastasis (78).

BA promotes gastric cancer malignancy through the cross-talk of multiple pathways, and its effects are closely related to BA type and microenvironment. In the future, it is necessary to combine single-cell technology with targeted intervention strategies to realize precision therapy.

##### Colorectal cancer

An essential step in colon cancer cell transformation is the activation of epithelial-mesenchymal transition (EMT). At this stage, epithelial cells acquire mesenchymal cell traits, which enhance cancer cell movement and migration. WNT signaling is involved in EMT, and FXR inhibits WNT/β-catenin signaling activity in colon cancer cells. Activation of WNT signaling leads to increased expression of β-catenin in cells, and overexpression of FXR forms a complex with β-catenin, destabilizing the β-catenin/transcription factor 4 (TCF4) complex and repressing the transcriptional activity of WNT-related target genes. Conversely, it also antagonizes the FXR/RXRα complex and its transcriptional activity. SHP has also been found to act as a partial tumor suppressor by inhibiting the expression of cell cycle protein D1 and C-C motif chemokine ligand 2 (CCL2) due to FXR-mediated transcriptional activation of SHP, which reduces tumor cell proliferation and invasion to a certain extent (81).

As an agonist of FXR, obeticholic acid (OCA) is nearly 100 times more potent than CDCA, the natural ligand of FXR.OCA inhibits the activity and growth of HT-29 and Caco-2, and retards the proliferation of colon cancer cells by inhibiting the G1/S transition and inducing apoptosis. In the same process, EMT is also inhibited (82).

Recently, it has been found that the interaction of β-catenin and FXR impacts the antitumor effect of OCA on CRC cells. It is the level of β-catenin rather than its transcriptional activity that influences the antitumor effect of OCA. The combination of the two drugs increased the rate of apoptosis and the percentage of G0/G1-phase cells, but decreased the percentage of S-phase cells and also impaired the invasive capacity of cancer cells compared to single administration. In conclusion, the combination of OCA and nitazoxanide (NTZ) was more effective in CRC, and NTZ compensated for the deficiencies of OCA to some extent (83).

The FXR/miR-135A1/CCNG2 axis may be a critical therapeutic target for CRC. Numerous studies have shown that FXR is an effective inhibitor of tumor progression, and many FXR agonists can enhance the activity of FXR. Perhaps, produce good therapeutic effects in terms of its molecular mechanisms involved in the pathogenesis of CRC (84).

The major alterations in bile acid profiles or modifications in key diseases, involved receptors, and potential therapeutic strategies are summarized in **Table 2**.

**Table 2:** Alterations in bile acid profiles/modifications in key diseases and potential therapeutic strategies.

Disease	Bile acid change	Main receptor involved	Potential therapeutic strategy
Inflammatory bowel disease (IBD)	↓ BSH activity, ↓ 7α-dehydroxylation, acidic fecal pH	FXR, TGR5	FXR agonists, secondary bile acid supplementation
Obesity/Type 2 diabetes	Altered ratio of 12α-hydroxylated bile acids	FXR, TGR5	TGR5 agonists (GLP-1 secretion)
NAFLD/NASH	Reduced bile acid pool size, diminished FXR signaling	FXR, TGR5	FXR/TGR5 dual agonists
Hepatocellular carcinoma (HCC)	Accumulation of hydrophobic BAs (DCA, LCA); dysregulated FXR/TGR5 signaling	FXR, TGR5	FXR agonists (preclinical); TGR5 modulators; BA-lowering strategies
Colorectal cancer (CRC)	↓ FXR expression, ↑ Wnt/β-catenin signaling	FXR	FXR agonists (e.g., obeticholic acid)
Gastric cancer (GC)	Increased secondary bile acids, NF-κB activation	TLR4, NF-κB	Reduce bile reflux, target inflammation

#### AI and multi-omics in bile acid modification research

Recent advances in artificial intelligence (AI) and multi-omics integration have accelerated the discovery and functional annotation of novel bile acid modifications. Machine learning models, including random forest and deep learning, predict substrate specificity of microbial enzymes (e.g., BAS-suc) and identify novel modification sites at C3 and C24 (85).Structure-based AI combined with molecular docking (e.g., AlphaFold, AutoDock-Vina) accurately predicts binding affinity and signaling bias of modified bile acids toward FXR and TGR5, enabling rational design of selective TGR5 agonists. Multi-omics integration (metabolomics, metagenomics, transcriptomics) combined with AI network analysis reveals the microbiota–bile acid–host metabolism axis and identifies disease-specific bile acid markers (e.g., DCA-3S, 3-sucCA) (86).Recent examples include AlphaFold-derived structural models of microbial BA-modifying enzymes, and machine learning pipelines that screen TGR-biased bile acid ligands from large compound libraries (87, 88).AI-driven strategies bridge basic bile acid chemistry and clinical translation, facilitating personalized metabolic therapy.

## Conclusion

Recent advances in bile acid modifications, including 3-acylation, C24-amidation, and polyamine conjugation, have unveiled the intricate interplay between host metabolism and gut microbiota. These structural alterations profoundly impact metabolic diseases (e.g., MASH, diabetes), cancers, and immune disorders through multiple mechanisms: (1) remodeling nuclear receptor signaling (FXR, TGR5); (2) modulating gut microbiota composition; and (3) restoring intestinal barrier integrity. Notably, 3-succinylcholic acid (3-sucCA) demonstrates therapeutic potential by enhancing probiotic colonization in steatohepatitis, while polyamine-conjugated bile acids regulate T cell differentiation to suppress intestinal inflammation. Furthermore, sulfation-mediated detoxification and localized acyl esterification offer targeted therapeutic approaches for bile acid pathway modulation.

BA modifications demonstrate both tissue-specific localization and systemic functions, integrating metabolic and immune regulation via the gut-liver axis. Specifically, microbial-derived 3-succinylcholic acid (3-sucCA) translocates hepatically via portal circulation, suppressing Kupffer cell activation and alleviating metabolic dysfunction-associated steatohepatitis (MASH). Future research directions should focus on deepening molecular mechanisms, resolving the three-dimensional structure and catalytic mechanism of novel modified enzymes (e.g., BAS-suc), and developing highly selective inhibitors or agonists; grasping precise drug design to optimize tissue specificity (e.g., hepatic-targeted FXR agonists) and reduce systemic side effects; and practicing multiorganomics integration by combining single-cell sequencing, metabolomics and spatial mass spectrometry imaging to reveal the spatiotemporal dynamics of bile acid modification and its trans-organ regulatory network; and clinical translational validation to advance trials of diagnostic markers (e.g., DCA-3S) and therapeutics (e.g., phage targeting of pathogenic bacteria) based on modified bile acids. With technological innovations (e.g., click chemistry enrichment, CRISPR screening) and interdisciplinary collaborations, bile acid modification research will accelerate the leap from basic science to clinical medicine, opening up new pathways for personalized medicine.
