# Supplementary figures and images for: Bile acids and bile acid modification in health and disease: from novel modifications to therapeutic interventions
Source: Front Endocrinol (Lausanne). 2026 Jun 4;17:1837504. doi: 10.3389/fendo.2026.1837504 (PMC13275213; doi:10.3389/fendo.2026.1837504)

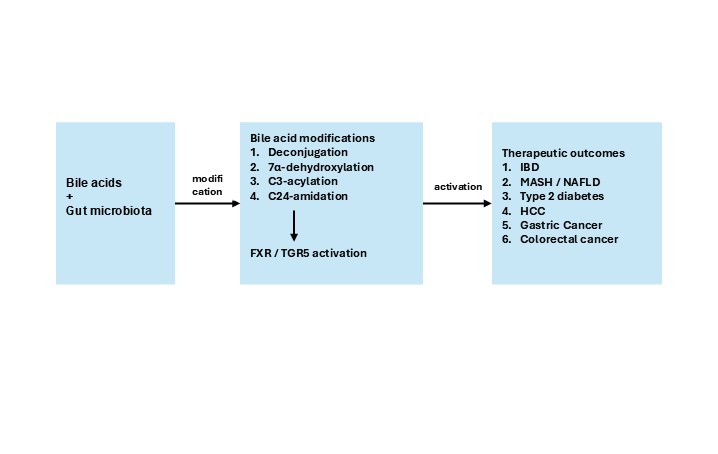

Supplement: Supplementary file 1 [file Image1.jpeg]
